# Extensive Craniocervical Abscess after Transoral Ganglionic Local Opioid Analgesia at the Superior Cervical Ganglion for Atypical Trigeminal Neuralgia: Report of a Severely Complicated Case

**DOI:** 10.1155/2018/5247594

**Published:** 2018-05-24

**Authors:** Christoph Sproll, Bernd Turowski, Rita Depprich, Norbert R. Kübler, Marion Rapp, Julian Lommen, Henrik Holtmann

**Affiliations:** ^1^Clinic for Oral and Maxillofacial Surgery, University Hospital, Heinrich-Heine-University, Moorenstraße 5, 40225 Düsseldorf, Germany; ^2^Institute for Diagnostic and Interventional Radiology, Division of Neuroradiology, University Hospital, Heinrich-Heine-University, Moorenstraße 5, 40225 Düsseldorf, Germany; ^3^Clinic for Neurosurgery, University Hospital, Heinrich-Heine-University, Moorenstraße 5, 40225 Düsseldorf, Germany

## Abstract

Ganglionic local opioid analgesia (GLOA) describes the application of low-dose opioids close to sympathetic as, for example, to the superior cervical ganglion. GLOA can be effective in different pain syndromes affecting the head and face region and has been considered to be a safe technique with few complications reported so far. We present the case of a patient who received a single, transoral GLOA for a refractory trigeminal neuralgia. The patient subsequently developed an extensive epidural abscess at the craniocervical junction, requiring ultimately transoral odontoid resection and dorsal stabilisation. This severe complication challenges the role of transoral infiltration therapies in analgetic medicine.

## 1. Introduction

Initial reports on the effectiveness of morphine locally administered to sympathetic ganglia were published thirty years ago [1]. The term ganglionic local opioid application (GLOA) for this procedure was coined in 1986 by Sprotte [2], and to date, GLOA is an accepted technique, especially in Europe.

For GLOA, opioid sometimes combined with a local anesthetic agent is dissolved in physiologic saline solution (e.g., 0.03 mg buprenorphin in 3 ml saline solution). Afterwards and under direct visual control, the sitting patient gets transorally an injection with an atraumatic spinal needle retrotonsillary into the lateral pharyngeal wall at the height of the 2nd cervical vertebra. Preinterventional oropharyngeal disinfection complies with those for oral surgical interventions (chlorhexidine, octenidine oder phenole). By using a spacer, one can assure an infiltration of the needle at a maximum of 10 mm. Special attention should be put on the anatomical proximity to nervus vagus, nervus glossopharyngeus, nervus hypoglossus, and nervus laryngeus superior as well as to arteria carotis interna [3–5].

Since its introduction for pain treatment many years ago, predominantly only several small cohort studies/case series were conducted to analyze the benefits of this method as compared to local anesthetic sympathetic chain blockade and/or intravenous regional sympathectomy (IVRS) in sympathetically maintained pain (SMP) [1], trigeminal neuralgia [2, 4], postherpetic neuralgia, chronic facial pain, complex regional pain syndrome (CRPS) [6, 7], postoperative glossopharyngeal neuralgia [8], and phantom pain [9]. The clinical effects of GLOA can be divided into immediate, intermediate and long-term effects. The short-term analgetic effects occur no later than 20 minutes upon injection and are more distinct in patients with zoster or trigeminal neuralgia. After one month of repetitive GLOA treatment, the level of continuous pain is reported to decline significantly in responders [4]. The long-term effects of GLOA are only documented by few studies but relate distinctively with the immediate effects. In the analysis of Elsner and colleagues, 21% of the patients remained free of pain after three years and the majority of the remaining patients reported significant decrease in pain duration and severity [10]. Within previous studies, GLOA was applied concomitantly to multiple conventional treatment options; therefore, the sole effect of GLOA remains to be elucidated especially when looking on actual studies that found no distinguishable effect comparing GLOA with the saline/placebo injection [11]. In a study conducted by Goebel et al., it was deemed unlikely that a single GLOA injection with buprenorphine reduces symptoms of chronic pain when compared to a placebo saline injection [12]. Thereby, the efficacy of GLOA is still and up to now questioned.

GLOA has been reported with virtual absence of major complications up to now. Among the minor complications, primarily, accidental intravasal injection of the buprenorphine is the most common [3, 5]. Additionally, respiratory depression, sedation, somnolence, nausea, vomitus, circulatory collapse, and distinct miosis have been reported. Local pain at the injection site, short-term muscular tension in the neck, vertigo, xerostomia, *β*_2_-mimetic-sensitive bronchoconstriction [10], dysphagia, and transient hoarseness are rather uncommon effects [4]. The absence of major complications associated with the procedure to date may be caused in the exercise of GLOA not only as a therapeutic, but also as a diagnostic tool in the management of different neuropathic pain syndromes.

However, this is challenged by the following first case presentation of a patient with atypical trigeminal neuralgia who developed a severe complication after a single GLOA procedure.

## 2. Case Presentation

**Figure 1:**
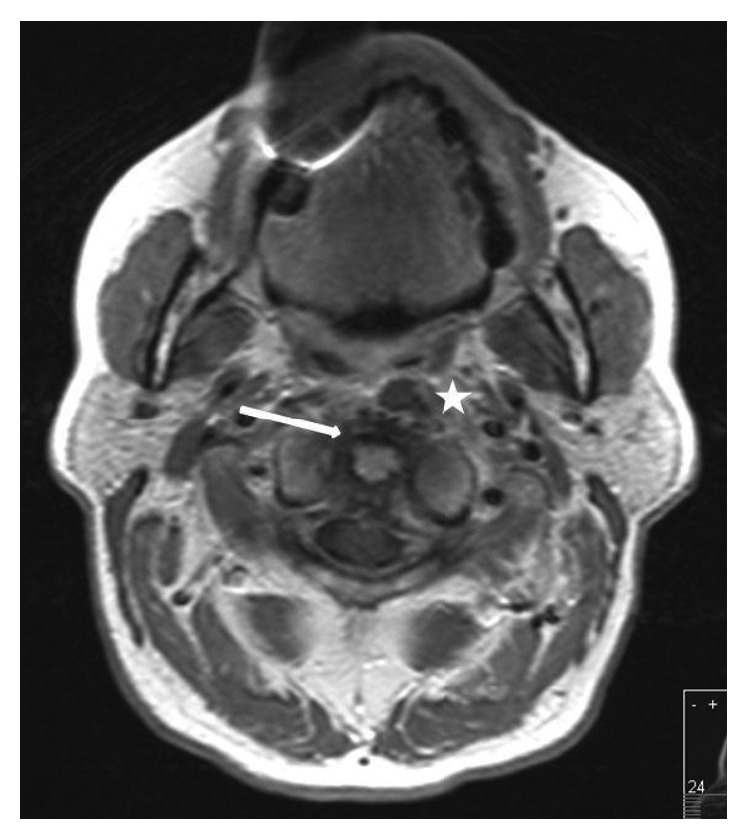
Axial MRI slice at initial presentation: contrast-enhanced T1-weighted axial MRI scan at the level of the dens axis. The white arrow identifies an annular enhancement round the dens axis while the white asterisk indicates the focus ventromedial of the left arch of the Atlas with 11 × 8 mm in diameter.

A 68-year-old female patient was treated for several months in a dental practice for craniomandibular dysfunction (CMD) with an occlusal splint, physical therapy, and pain medication. Initially, she rated the pain as 80/100 on a visual analogue scale (VAS), but after a 3-month period of conservative treatment, a significant pain reduction to a value of 20 was achieved. Subsequently, she reported stabbing, electric shock-like pain in the left mandibular region triggered by mastication at follow-up examination four weeks later. Secondary diagnoses to name were only a hypothyreosis (known for 15 years) and anarterial hypertension (diagnosed 10 years before). Under the clinical suspicion of triggered atypical trigeminal neuralgia by an external oral and maxillofacial surgeon, she received one transoral GLOA at the left GCS (ganglion cervicale superius) in a private oral and maxillofacial practice without using an analgetic specific medication (without a documented reason) before. One week later, the patient developed a decreased rotational mobility in the cervical spine and was therefore referred to a local general hospital, where she got an X-ray of the cervical spine (normal finding) and was treated by a cervical collar and analgetic therapy. Due to a further worsening of her condition the following three days, she was sent to our clinic in a significantly reduced general condition, with cervical and retroauricular pain, odynophagia, and hoarseness. Clinical neurological and orthopaedic council examinations revealed a painful loss of mobility (rotation and lateral bending) in the cervical spine, tenderness on palpation around the cervical spine, fever (39.5°C), an intact sensorium in the head and neck region and the upper extremities, no meningismus, absence of Lhermitte and Lasegue's signs, and no latent or manifest paresis in all four extremities. An intraoral investigation revealed para- and retropharyngeal moderate tissue swelling. Blood tests showed elevated C-reactive protein levels (CRP) (29.4 U/ml) and leucocytosis (white blood cell count (WBC) 13,700/*µ*l) as the most important findings. Magnetic resonance imaging (MRI) displayed pathological constrast enhancement in the middle cranial fossa, mainly around left Meckle's cave, extending anteriorly and, along the clivus caudally to the C2 level. Further, the dens axis displayed a small circular enhancement that caused a slight compression of the dural sac without significant myelon compression (Figure 1). Contrast enhancement was also seen along the ventral cervical muscles on the left side as well as a focus ventromedial to the left arch of the Atlas with a diameter of 11 × 8 mm and a clear circular enhancement. Hence, an extensive and phlegmonous craniocervical infection originating from the area of the superior cervical ganglion was diagnosed.

On the same day, an incision of the retropharyngeal abscess was performed, and an intravenous (i.v.) antibiosis with ampicilline and sulbactam was initiated. During the next two days, the clinical condition of the patient improved significantly and CRP counts decreased to 12.1 U/ml. Microbiological testing revealed colonies of Enterobacteriaceae and Micromonas micros as well as Peptostreptococcus and *Veillonella* species. Antibiosis was therefore changed to meropenem (3 × 2 g i.v. per day). Four days after initial abscess drainage, the patient complained of increasing pain on rotation of the head and an increase of the infection parameters was seen again (CRP: 17.7 U/ml and WBC: 19.900/*µ*l).

**Figure 2:**
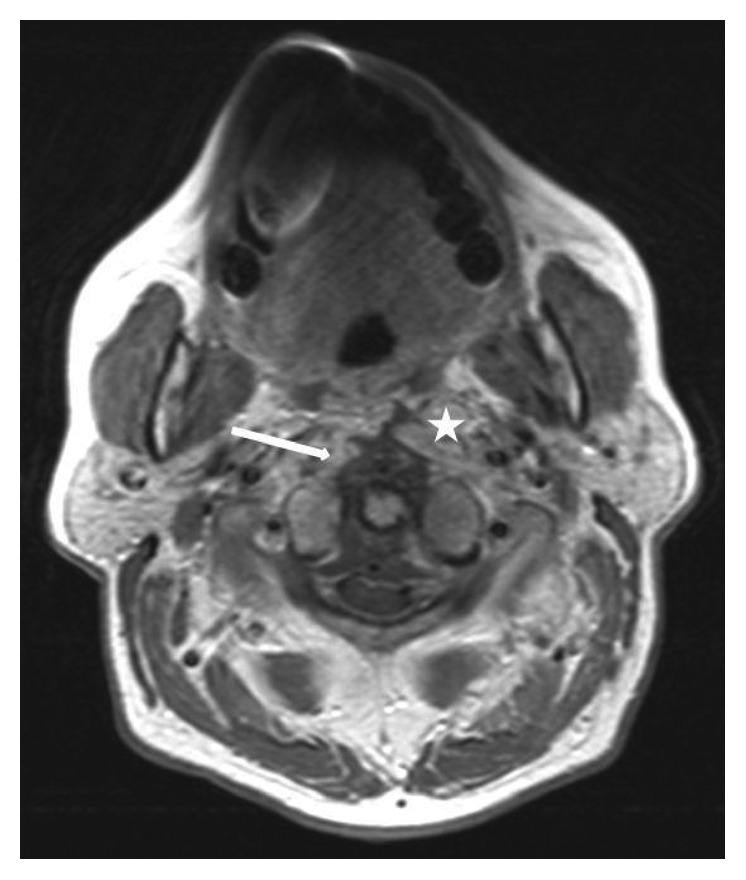
Axial MRI slice five days after incision and drainage of the prevertebral abscess: contrast-enhanced T1-weighted axial MRI scan shows the incised abscess cavity (white asterisk) which is connected to a progressive fluid retention especially in the area between the dorsal ligament and the ventral dura with a slight compression of the myelon (white arrow). This epidural abscess ranged from the foramen magnum to the upper ridge of the third cervical vertebra.

**Figure 3:**
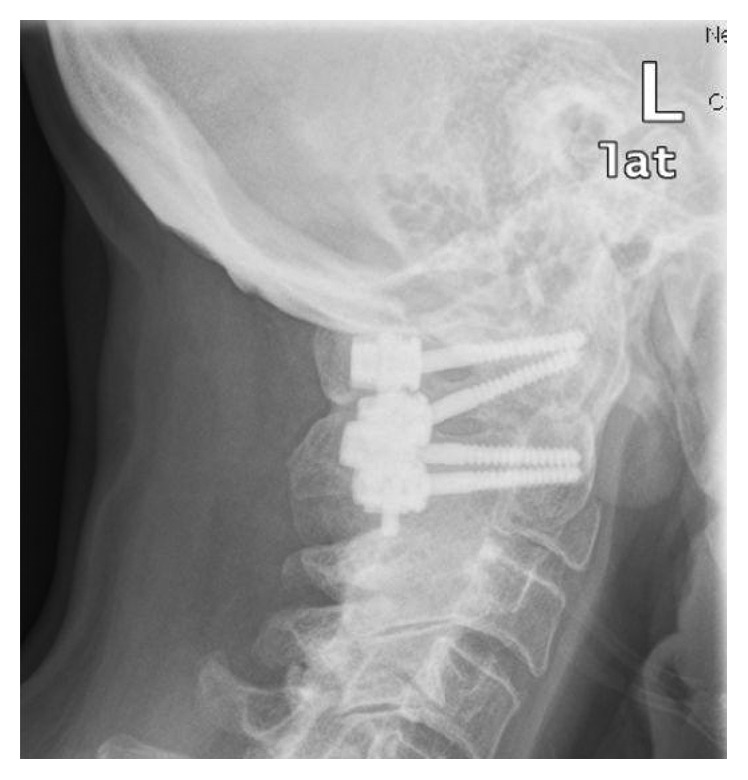
Lateral X-ray of the neck after dorsal stabilisation. The inserted devices are in correct position in all plains.

The follow-up MRI, five days after the initial diagnosis, displayed a significant progression in terms of a paravertebral and intraspinal abscess formation in the upper cervical spine (Figure 2). The retropharyngeal and paravertebral phlegmonous infection compromised the cervical myelon from the dorsolateral region and extended from the C0 to C2 level. Additionally, the epidural abscess in the dorsal aspect of the dens axis had also progressed, resulting in an odontoid ostitis. Furthermore, the incised retropharyngeal abscess cavity was connected by a small fistula to a second cervical abscess, medial to the sternocleidomastoid muscle. Due to increasing fever, pain, and progressing loss of vigilance, the patient underwent a removal of the ventral aspect of the C1 arch, odontoid, and related ligaments through a transoral approach. Intraoperatively, the abscess did not penetrate the dura, and an epidural drain was applied for daily irrigation and fluid drainage. The cervical abscess medial to the sternocleidomastoid muscle was drained. The microbiological analysis revealed sporadic *Candida glabrata*, very few colonies of *Streptococcus constellatus* and sporadic mixed flora, and the i.v. antibiotic and antimycotic therapy was changed to clindamycin (3 × 1200 mg), ampicillin and sulbactam (3 × 3 g), and voriconazol (2 × 200 mg). Eight days after the odontoid resection, the infection parameters returned to nearly normal values (CRP 1.8 U/ml and leucocytes 7.200/*µ*l), and posterior C1-C2–instrumentated fusion was performed (Summit™, DePuy) to restore craniocervial stability (Figure 3). Weaning from ventilation and the further course were uneventful, and four weeks after admission, the patient was transferred to a rehabilitation facility with only minor disturbances of orientation. On follow-up examination four months later and till today, the patient was continuously seen with no neurological deficit and in good clinical condition.

## 3. Discussion

The desire of patients suffering from chronic orofacial pain syndromes (i.e., typical and atypical trigeminal neuralgia) for immediate pain relief has led to the introduction of first-line treatment options including drug therapy with anticonvulsants or analgetics (i.e., carbamazepine and oxacarbamazepine) and injection of local anesthetics close to sympathetic ganglia [13]. Beginning in 1980, ganglionic local opioid analgesia (GLOA) started to gain importance in Europe [14]. Due to the absence of serious adverse side effects reported so far, and to a notable decrease in pain intensity in responders even after a single injection, GLOA was established as a treatment alternative in some cases of orofacial pain syndromes.

Herein, we provide the first report on a severely complicated course after a single GLOA at the left GCS with a hospital stay of four weeks, six surgical procedures, and a two-month stay in a rehabilitation clinic required for recovery. Notably, the pain prompting the GLOA treatment had resolved after one single application.

Due to the aforementioned alternative treatment options, the course of our patient challenges the role of GLOA as a diagnostic and therapeutic tool in analgetic medicine especially for atypical TN (especially when no conservative analgetic treatment pathway was performed before any invasive method at all).

Talking about chronic orofacial pain syndromes and their therapies, it means that only well-established ablative and nonablative procedures, supported by clinical practice guidelines, display the highest degree of long-term pain control and the lowest therapy-associated morbidity. Nevertheless, even these techniques should be reevaluated in an ongoing process to detect possible adverse effects like in this case.
